# Identification and Analysis of Genes and Pseudogenes within Duplicated Regions in the Human and Mouse Genomes

**DOI:** 10.1371/journal.pcbi.0020076

**Published:** 2006-06-30

**Authors:** Mikita Suyama, Eoghan Harrington, Peer Bork, David Torrents

**Affiliations:** 1 European Molecular Biology Laboratory, Heidelberg, Germany; 2 Max Delbrück Center for Molecular Medicine, Berlin-Buch, Germany; Universitat Pompeu Fabra, Spain

## Abstract

The identification and classification of genes and pseudogenes in duplicated regions still constitutes a challenge for standard automated genome annotation procedures. Using an integrated homology and orthology analysis independent of current gene annotation, we have identified 9,484 and 9,017 gene duplicates in human and mouse, respectively. On the basis of the integrity of their coding regions, we have classified them into functional and inactive duplicates, allowing us to define the first consistent and comprehensive collection of 1,811 human and 1,581 mouse unprocessed pseudogenes. Furthermore, of the total of 14,172 human and mouse duplicates predicted to be functional genes, as many as 420 are not included in current reference gene databases and therefore correspond to likely novel mammalian genes. Some of these correspond to partial duplicates with less than half of the length of the original source genes, yet they are conserved and syntenic among different mammalian lineages. The genes and unprocessed pseudogenes obtained here will enable further studies on the mechanisms involved in gene duplication as well as of the fate of duplicated genes.

## Synopsis

The duplication of genes is considered one of the major sources of biological diversity, as it provides the necessary conditions for the generation of new gene types and functions. Even though, after a gene is duplicated, one of the copies normally undergoes inactivation, it can eventually establish in the genome as a novel gene with new functionality. The identification of the molecular basis of gene duplication and the forces that determine the fate of the resulting copies is essential to understand how genes and, ultimately, organisms evolve. The first step in this direction is the identification of duplicated genes and pseudogenes, which still remains a challenge for standard procedures of automated genome annotation. The authors have developed a methodology that comprehensively identifies and classifies these regions, and provide the collections of duplicated genes and pseudogenes found in the human and mouse genomes. Among these, there are 420 previously unidentified potentially functional genes, which include examples of partial duplicates with less than half of the length of the original source genes. Furthermore, they also provide preliminary novel biological insight into the mechanism of gene duplication, which will constitute the starting point for further studies of the fates and evolution of duplicated genes.

## Introduction

Gene duplication is the major source of biological innovation and diversity as it provides the necessary conditions for the appearance of new or more specialized protein functions [1]. In eukaryotic genomes, there are two major mechanisms through which coding gene regions duplicate: retrotransposition and non-homologous recombination. Whereas retrotransposition can lead in rare occasions to a functional mRNA copy [2], it usually results in processed pseudogenes. The present study focuses on gene copies that, on the other hand, arose through non-homologous recombination, which produces intact (unspliced) genes copies. It is generally agreed that after such gene duplications, there is a period of functional redundancy and, consequently, a partial relaxation of their associated selective constraints (for review see [3,4]). This allows each copy to accept a higher level of sequence modification and, therefore, explore new or more specialized roles as long as the basic ancestral function is not compromised. Although this situation can eventually lead to the formation of novel genes, it is generally believed that it normally ends with the silencing of one of the copies by the accumulation of lethal mutations, and the preservation of the other with the same (or eventually enhanced) basic ancestral function [5]. Non-functional paralogs are then expected to accumulate mutations at a neutral rate and degenerate as unprocessed pseudogenes. Similarly, apart from duplicated exons that lead to alternatively spliced isoforms [6], incomplete duplications of genes that can neither be transcribed nor translated into complete and functional proteins are also expected to undergo neutral degeneration right after their formation, as occurs with the vast majority of processed pseudogenes.

Currently the silencing of genes after duplication is poorly understood. Its frequency has been indirectly inferred either through theoretical approaches [7,8] or from the study of functional genes exclusively [5], without taking into account the population of dead gene copies, probably due to the lack of consistent annotation for these regions in public databases. Not only the identification of unprocessed pseudogenes, but also the overall identification and classification of independent gene copies within regions that underwent several rounds of tandem duplications, are not completely solved, as exemplified in a detailed analysis of a particular region of human Chromosome 2 [9]. Previous global analyses of dead gene copies in mammals have focused mainly on retrotransposed (processed) pseudogenes [10–13], which appear to be far more abundant and easier to detect than unprocessed pseudogenes. We have already attempted to define collections of unprocessed pseudogenes in the context of a genome-wide identification of intergenic pseudogenes from several sequenced genomes [10,12–15]. The estimated number of these regions fluctuated significantly within mammals: between 3,000 and 4,500 per genome. However, on the basis of our recent and more detailed analysis of the finished human Chromosomes 2 and 4 [16], we estimate that the human genome might actually contain no more than 2,000 unprocessed pseudogenes, because previous sets were somewhat inflated by misclassified retrotranscribed (processed) pseudogenes. In addition to these large-scale approximations, several hundred unprocessed pseudogenes also have been identified during the annotation of single human chromosomes (available from VEGA database, [17]) and from detailed studies focused on particular gene families or genomic regions (e.g., [12,13,18–21]). Despite all these efforts, a considerable fraction of human and mouse unprocessed pseudogenes is likely to be unannotated or incorrectly classified owing to the difficulties in analyzing complex regions with multiple copies of genes.

Using filtering procedures performed on the available assemblies of the human and mouse genomes, we have carried out a consistent and comprehensive search for gene duplicates independent of previous gene annotations. We have distinguished the potentially active from the non-functional copies in order to construct the first reliable set of unprocessed pseudogenes.

## Results

### Identification of Human and Mouse Duplicates with Coding Potential

For the detection and prediction of gene duplicates in human and mouse, we first identified all regions with coding potential (i.e., protein coding genes and derived copies). To obtain a reliable set, we required the presence of a homolog in existing sequence databases. The respective search methodology had been previously applied to the identification of non-functional gene duplicates between annotated functional genes of different metazoan genomes [10,12–16]. In contrast to the previous procedure that took for granted the available annotation of the gene sets, we performed de novo gene predictions independent of current gene annotations. Using the comparison of all known protein sequences against the entire human and mouse repeat-masked genomes, we identified and reconstructed the putative coding sequence of 43,247 and 43,715 regions in each organism respectively (see Materials and Methods), which are likely to correspond mainly to genes and to the two fundamental types of pseudogenes: retrotransposed (processed) and duplicated (unprocessed). We next assessed the completeness of these sequence collections and, in turn, the overall sensitivity of our underlying search protocol in the context of existing genome annotations. For this we compared our sequence collections with the most widely used genomic databases, which, despite their differing methods, are expected to cover practically all human and mouse genes, and a fraction of human pseudogenes (Table 1, first column). From these comparisons, we first concluded that our method identified nearly all of the human and mouse coding genes, as we found overlap with 96% and 94% of the human functional regions included in the RefSeq [22], and ENSEMBL [23], ENCODE [18], and VEGA [17] databases, and with the 87% and 94% of the ENSEMBL and RefSeq mouse genes, respectively. Our coverage of pseudogenes was similarly high, our human predictions detecting between 80% and 94% of all processed and unprocessed human pseudogenes annotated in the ENCODE, VEGA, and “YALE” [11] databases.

**Table 1:**
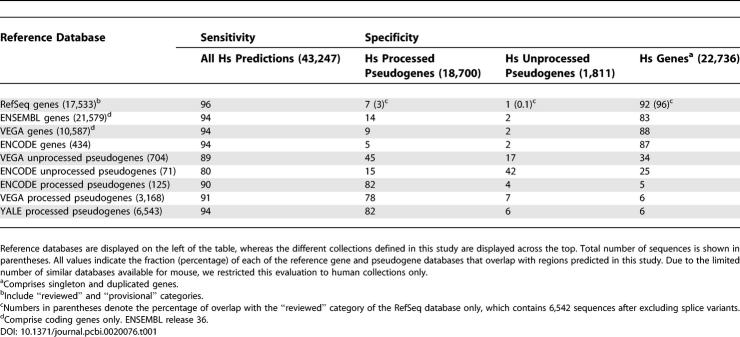
Assessment of the Identification and Classification Procedures

We also assessed the accuracy of our predictions by focusing on three aspects of the predicted genomic structure that are of particular relevance for the subsequent analysis of the data: (1) the accuracy with which we distinguish each independent (pseudo)gene, i.e., the amount of fragmentation and fusion, which could be particularly frequent in complex regions with multiple gene copies; (2) the coverage of the coding region; and (3) the level of artificial insertion of truncations (in-frame stop codons or frameshifts). This also reflects the accuracy at which intron–exon boundaries are defined, since usually, incorrectly predicted splice sites give rise to artefactual truncations downstream. As the quality of any homology-based prediction of genes and pseudogenes depends strongly on the degree of similarity between the known protein sequence used as a template and the target genomic region, we performed this test by carrying out a second and completely independent round of predictions (a jackknife test) considering only genes coding for reviewed RefSeq cDNAs, taking care not to use their corresponding protein products as a reference (see Materials and Methods for details). In this test, each of the predictions was then directly compared with the real RefSeq protein product of that gene. The results of this test were overall satisfactory: (1) Only 6% of the predictions were either fragmented or fused to a neighboring gene (please note that in order to avoid the overestimation of duplicated (pseudo)genes, our procedure deliberately fuses terminal duplicated exons to the preceding complete gene, as these are frequent in the human genome [6] and can be involved in the formation of alternatively spliced isoforms rather than constituting independent and incomplete gene copies); (2) we also found that as many as 90% of our predictions of RefSeq genes covered more than 90% of the known coding regions; and (3) no more than 4% of them contained artefactual stop codons or frameshifts.

Therefore, from the comparison of all our predictions with available and representative gene and pseudogene collections, and the independent evaluation of their (pseudo)coding regions, we conclude that the overall performance of the identification and prediction procedures is sufficiently high to use the resulting data as a basis for the identification and analysis of duplicated genes.

### Assignment of Orthology and Exclusion of Processed Pseudogenes

**Figure 1:**
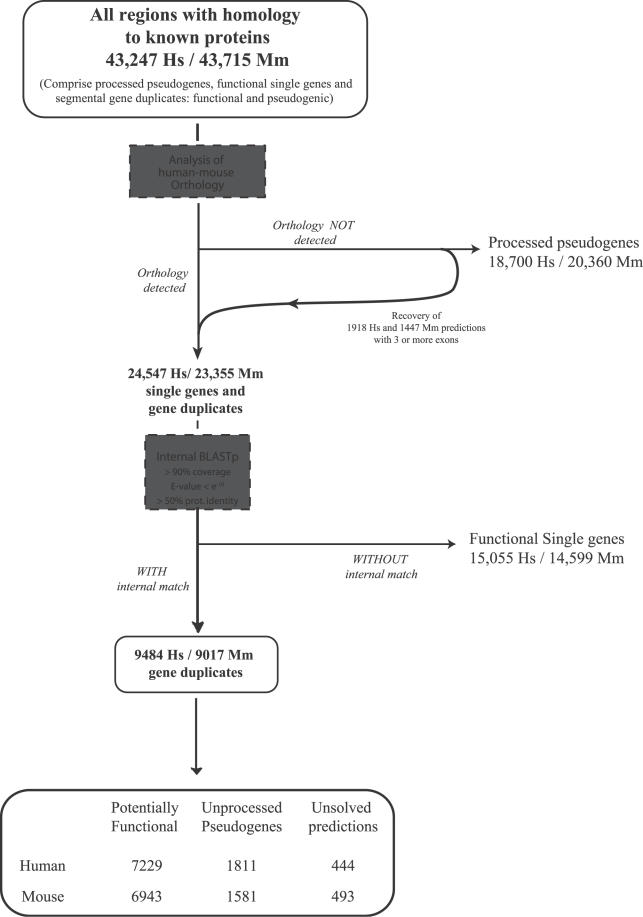
Schematic Representation of the Procedure Employed to Classify the Three Major Types of Genes and Derived Sequences Identified in Human and Mouse According to Their Origin between and within Each Species Dashed boxes denote key action steps in the procedure. See text for details.

A next step in defining duplicates, in particular those that have emerged after the human–mouse split, is to assign orthology between the 43,247 human and 43,715 mouse candidate genes and pseudogenes. These sets should at this stage consist of a mixture of processed pseudogenes, single functional genes, and both functional and pseudogenic gene duplicates. To distinguish between these, we performed a series of sequence comparisons of all the candidates, both between and within each of the species (Figure 1). By evaluating human–mouse reciprocal sequence similarity and genomic context, we could identify orthologous relations for 22,629 human and 21,908 mouse gene predictions. Of these relations, 16,890 arose directly by human–mouse best reciprocal matches mostly in syntenic regions, i.e., that correspond to 1:1 orthologs. Interestingly, the fact that among these clear orthologs, only 300 (1.7%) appear to be truncated in human, and of those, only 65 were also found truncated in mouse, confirms that practically no pseudogenes formed in the human–mouse ancestor are still conserved at the level of sensitivity used in this and other studies. The remaining “non-1:1” orthologs represent more complex orthologous relationships, most likely involving gene families that underwent expansions in one or both of the lineages. On the other hand, 20,618 human and 21,807 mouse predictions did not show any sign of orthology and were initially assumed to correspond mostly to non-functional retrotransposed gene copies, i.e., processed pseudogenes, which are expected to be located far from their parental genes. These sets are also expected to include genes that either have been translocated in one of the genomes after the human–mouse split, and a few whose corresponding ortholog was lost completely from the other genome. A fraction of these genes was detected by analyzing the intron–exon composition of all predictions with no assigned orthology. This was achieved by selecting those sequences that were predicted with two or more introns and are subsequently incompatible with a retrotranspositional origin. Please note that allowing the presence of an intron for processed pseudogenes might be a restricted criterion, but it is also a conservative one because real processed pseudogenes might often contain insertions of foreign DNA that can be interpreted as introns during the final steps of our prediction pipeline (i.e., by GENEWISE). A total of 1,918 human and 1,447 mouse predictions with no detectable orthology were found to present two or more introns and were therefore reclassified as potential genes or unprocessed pseudogenes. By extrapolating the portion of functional genes (from RefSeq) that have fewer than two introns, we finally estimated that around 300 genes and unprocessed pseudogenes (that were recently translocated or that lost their corresponding ortholog) remained undetected and hence misclassified as processed pseudogenes. Although the total number of human processed pseudogenes defined here is consistent with previous estimations of around 20,000 made by independent studies [11], it is considerably larger than in early studies (around 14,000 [10]). This difference could be partially explained by a slight increase of sensitivity during the present homology search (i.e., the number of annotated known proteins in databases has increased since then), or by the fact that this study did not rely on gene sets (e.g., ENSEMBL) that have varied dramatically in the past years and were known to include a significant number of processed pseudogenes [12,13,24]. But, above all, the refinement in the detection of orthology and the consequent improvement in the classification of pseudogenes, rather than the identification of new regions, is what largely explains this increase in the number of processed pseudogenes.

Taken together, using orthology and exon composition, we could classify all 43,247 human and 43,715 mouse predictions, into 24,547 and 23,355 genes or gene duplicates, and 18,700 and 20,360 into processed pseudogenes, respectively. In order to assess the discriminatory power of the criteria used for this first step of our classification procedure, we again compared each of the resulting human collections with the available gene and pseudogene collections. In support of our method, we first observed that a low fraction of genes have been here misclassified as processed pseudogenes, as 1% of all of our 18,700 retrotransposed regions overlap in the genome with only 3% of the nearly 7,000 reviewed RefSeq genes (Table 1, second column). Despite remaining similarly low, this amount of overlap appears to be higher when considering other gene databases. This could be partially explained by the different levels of misannotated processed pseudogenes within these databases, since we observe that the degree of overlap tends to be higher in databases with lower levels of confidence: 5% of the ENCODE coding genes overlap with our processed pseudogenes, as do 7% of all (including non-reviewed) RefSeq regions, 9% of the VEGA coding genes, and 14% of the same type of ENSEMBL genes. We next observed that, as with the misclassification of genes as retrotransposed regions, the opposite events appear to be also infrequent, as 9% of the ENCODE, 13% of the VEGA and 12% the YALE processed pseudogenes have been considered here as genes or unprocessed pseudogenes (addition of the third and fourth columns of Table 1; see the text below for details on the discrimination between functional and pseudogenic gene duplicates). But, surprisingly, despite the low overlap (15%) of ENCODE unprocessed pseudogenes with our retrotransposed regions, we observed a significant discrepancy with the corresponding VEGA regions, which showed a 45% (319 regions) overlap with our processed pseudogenes. From a manual inspection of the alignment of each of these 319 VEGA predictions with their closest functional gene, we found that, although 141 of them clearly maintain at least one of the original introns, and therefore can be classified as real duplicated pseudogenes, the remaining 178 show either no detectable conservation of introns or clear evidence of intron loss, which is consistent with a retrotranspositional origin.

### Identification of Gene Duplicates

In order to identify duplicated (pseudo)genes among the remaining 24,547 human and 23,355 mouse candidate gene sets, we compared them with and in-between the two species. We recorded as close paralogs those pairs of sequences that matched significantly (BLAST E-value < 1e-10 and ≥50% sequence identity) over at least 90% of either of the sequences within a species. This last condition excludes the pairs of sequences that show similarity only at the domain level (e.g., shared SH3 domains). These constraints resulted in a total of 9,484 and 9,017 gene duplicates in human and mouse, respectively. The analysis of the genomic location of these sequence pairs shows a clear correlation with their relative age of formation: Paralogous relations with higher sequence identity are normally intrachromosomal, whereas older ones tend to involve different chromosomes (Figure S1). Of those pairs in which both sequences are located within the same chromosome, 81% are less than 1 Megabase apart, and more than half can be found even within a distance of 100 kilobases.

### Discrimination of Functional and Inactive Gene Copies

The genes in duplicated regions should be distinguishable from unprocessed pseudogenes by the integrity of functional features such as promoters and coding region. The activity of promoters is difficult to test, and indirect functionality measures via expression evidence (e.g., through the analysis of expressed sequence tags [ESTs]) are not straightforward because of mapping difficulties for close paralogs and additional translational control. Therefore, in addition to syntenic conservation, the integrity of the coding region is often used to test for functionality [16,21], i.e., the completeness of the gene and the presence or absence of truncations (in-frame stop codons, or frameshifts). Gene copies that possess truncations and/or incomplete coding regions are likely to be inactive, whereas those that are conserved in synteny between human and mouse, or are complete and uninterrupted are potentially functional.

**Figure 2:**
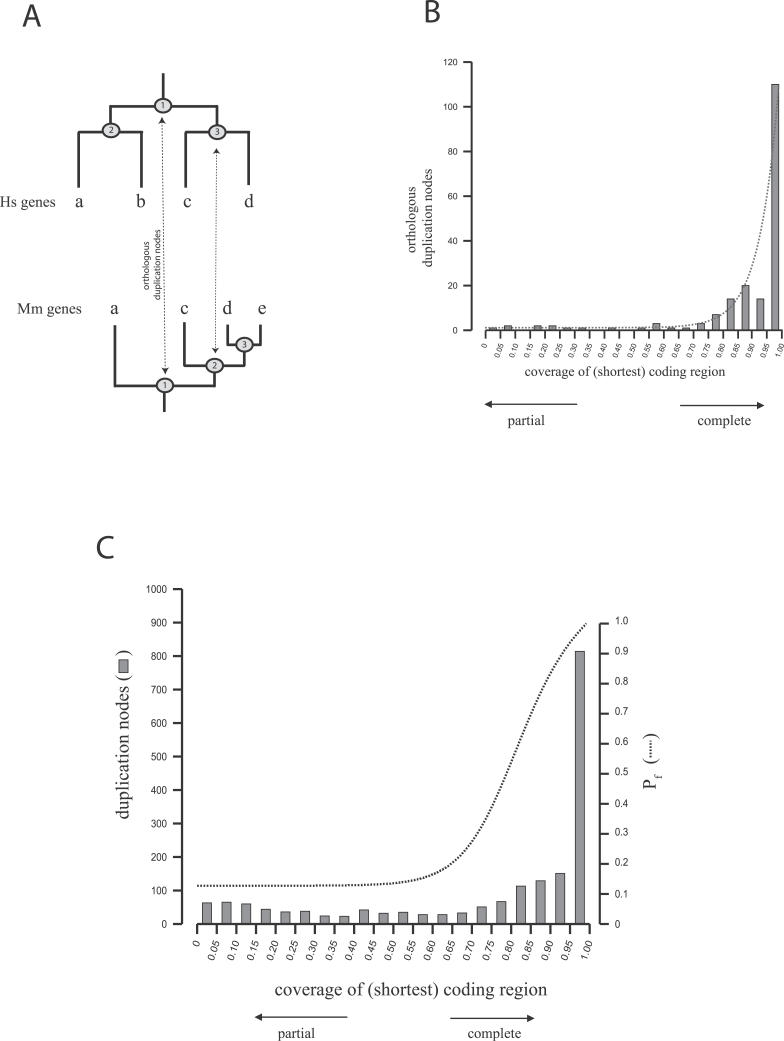
Analysis of Gene Coverage between Mouse and Human Paralogs (A) Identification of orthologous duplicated pairs. Genes are labeled with letters (same letters in human and mouse mean best reciprocal orthologs, e.g., genes “a,” “c,” and “d”). Numbers within circles in tree nodes represent gene duplication events. Dashed lines indicate orthology between human and mouse duplication nodes, which is inferred from the orthologous relations between the products of that duplication in each of the organism. (B) Distribution of orthologous duplication nodes in human according to the coverage of the shortest coding region relative to the longest one. The line corresponds to the exponential curve adjusted to the observed data (see Materials and Methods). (C) Distribution of the coverage of all duplicates found in human (columns), and probability for being functional according to the coverage (*P*_f_, dashed line).

Although the presence or absence of truncations is relatively easy to detect, there is little information about how complete a gene copy has to be in order to remain functional. Although it will vary from gene to gene, and incomplete copies coding for less than 50% of the closest paralog can retain functionality [9], we expect that duplicated coding regions usually require a minimal coverage in order to retain functional potential. These values can be estimated from the comparison of functional paralogs (i.e., gene copies that have survived a period of functional redundancy and are conserved in the population). Such cases were identified among the gene duplicates as pairs of paralogs that showed clear orthologous relationships between human and mouse because they have been kept in both genomes independently since they diverged over at least the last 75–100 million years ago (Figure 2A). The comparison of the relative length of these human functional paralogs shows that the majority have coding regions that can usually be well aligned over more than 80% of their length (Figure 2B). Even though this distribution suggests that only nearly complete gene copies remain functional, this might simply be the consequence of a higher tendency of genes to duplicate entirely as suggested by the overall predominance of complete gene copies in the genome (Figure 2C). In fact, the evaluation of the relative coverage of identical human gene copies, which have probably undergone a few deletion events, shows that at least 80% of all duplication events cover more than 70% of the ancestral coding region. Therefore, in order to estimate the probability of a gene duplicate being potentially functional (*P*_f_) according to the observed length of the coding region, we evaluated the length coverage of the longest coding region of all functional paralogous pairs normalized by the frequency at which such coverage is observed (see Materials and Methods). As shown in Figure 2C, this probability is high for complete or nearly complete copies, and decreases significantly for coding regions that cover less than 70% of the closest paralog, which is assumed to reflect the length of the ancestral gene.

### Incomplete Gene Copies Can Remain Functional

The probability of a duplicate being functional with an incomplete coding region is low but not zero. A total of 461 (231 in human and 230 in mouse) incomplete gene copies were found to present orthology and therefore suspected to be functional. A manual inspection of the copies that cover less than 50% of the neighboring paralogs but have an ortholog in the other species revealed that most of them are indeed partial gene copies that have been conserved in each of the two lineages. One example corresponds to a copy of the gene encoding the ETS-2 repressor factor *(ERF),* a gene that regulates proliferation of some types of cancer cell lines [25,26]. This partial copy, which has not been noticed by gene prediction programs or even during more detailed analyses of the region [27], comprises the second and third coding exons (i.e., covers 25%) of the *ERF* gene and contains a complete ESF-2 domain (Figure S2). The high degree of sequence identity of these two exons among the human, mouse, and the dog orthologs (between 90%–93% DNA identity in exons, whereas introns are too divergent to be aligned), and their low *d*_N_/*d*_S_ ratios (0.015, 0.022, and 0.018 for the human, mouse, and dog fragments, respectively) strongly indicate a selected function of the partial copy in mammals. Another example is a partial copy of the Sp5 transcription factor gene located in both human and mouse genomes on Chromosome 2. Both the human and mouse downstream orthologous copies of these genes, which comprise no more than 30% of the ancestral coding region, share 93% protein identity and contain a complete Zinc-finger domain. Among these orthologous partial copies, we have found other Zinc-finger–containing cases that have no homologous complete genes nearby, indicating that *Sp5* or homologous genes underwent several rounds of partial duplication and translocation events.

### Annotating Human and Mouse Unprocessed Pseudogenes

On the basis of the coverage-dependent probability of functionality derived above, the presence or absence of truncations, and orthology, we have initially classified the collections of 9,484 human and 9,017 mouse duplicates, respectively, into: (1) 7,229 and 6,943 *potentially functional* copies (genes) corresponding to coding regions that are non-truncated and more than 94% complete (*P*_f_ > 0.9), or with 1:1 clear orthologous relationships (operationally defined as best reciprocal matches in the global interspecies protein comparison); (2) 1,811 and 1,581 likely *non-functional* copies (or unprocessed pseudogenes) with truncated or incomplete coding regions (less than 60% of the length of their functional paralogs; associated to *P*_f_ < 0.2), and with no orthology; and (3) 444 and 493 of predictions without assignment *(uncertain)*, which comprises the remaining non-truncated coding regions between 60% and 94% paralog length coverage associated with inconclusive *P*_f_ values (between 0.2 and 0.9).

As a preliminary evaluation of the results of this classification, we compared some properties of the gene and pseudogene sets at different levels and observed: (1) Consistent with the nature of these sets, genes showed significantly higher (*p* = 3e-65) levels of expression: In human, 89% could be unambiguously assigned to ESTs (with an average of 136 ESTs per gene), in contrast to only 40% of the pseudogenic predictions (with an average of nine ESTs per region). Because the fraction of expressed pseudogenes could actually correspond to misclassified functional genes, we further evaluated whether the premature stop codons or frameshifts detected in these regions were also present in their assigned EST. We found that 75% of the premature truncations that are covered by ESTs (corresponding to 267 predictions) are in fact also present in the majority of the associated cDNAs. A fraction of the rest presented inaccuracies in the alignment, or included cases in which the EST sequence disagrees with the genomic sequence probably due to sequencing errors in either the cDNA or the genome, to posttranslational modifications, or to genomic polymorphisms, which also account for some of the cDNA-to-genome sequence disagreements found in other studies [28]. (2) We also compared human gene and pseudogene sets to known and predicted genes. The vast majority of functional regions, 94% in both human and mouse, overlapped with the corresponding RefSeq, ENSEMBL, VEGA, or ENCODE genes. The remaining non-overlapping functional fractions, which contain 222 predictions in human and 198 in mouse, correspond to potentially novel functional duplicated gene copies that have been overlooked by gene predictors (see supplementary data at http://www.bork.embl.de/Docu/human_mouse_duplicates). Among these, the majority have no clear ortholog in the other species, suggesting that they arose independently in each of the lineages. In order to keep our sets consistent with current annotated genes in each of these organisms, we further reclassified as functional genes the fraction of gene copies that overlapped with RefSeq and were initially labeled as unprocessed pseudogenes (344 human and 439 mouse) or uncertain (252 human and 213 mouse). A manual inspection of these regions indicates the presence of an important fraction of incomplete predictions that derived from templates annotated as partial proteins in RefSeq or SwissProt databases. Therefore, and in agreement with this comparison, we were finally able to respectively classify in the human and the mouse genomes 7,229 and 6,943 predictions as potentially functional genes, and 1,811 and 1,581 as unprocessed pseudogenes, respectively. The residual fraction that comprised 444 human and 493 mouse predictions remained unsolved with the criteria used here, and their classification likely requires additional strategies or even manual inspection.

In order to evaluate the level of agreement of this classification with current annotations, we have compared our human collections of genes (singletons and duplicated) and unprocessed pseudogenes with external databases. We observe a general agreement with the ENSEMBL database (83% and 2% of its genes overlap with our functional and pseudogenic regions, respectively; see third and fourth columns in Table 1), however we also found that a considerable fraction, 25% of ENCODE and 34% of VEGA duplicated pseudogenes, were classified as functional genes by our method. To clarify this disagreement, we carried out a detailed manual analysis of each of the 18 controversial ENCODE entries, which largely agree with the VEGA annotation. We found that, although these regions cover different types of functions, half of them correspond to a Chromosome 11 cluster of odorant receptors, a family of genes that has been always difficult to classify regarding functionality and origin, due to the fact that a considerable fraction of them have been silenced in several lineages [29], and they normally have only one coding exon. Even though we failed to identify expression evidence for the 18 ENCODE regions in EST databases, we believe that they should be considered preferentially functional because: (1) They present intact and complete open reading frames, some of which have been conserved since the human–mouse split, as they appear as clear orthologs to mouse regions (see above); and (2) several of the odorant receptors genes are expressed in fetal and adult tongue [30,31], which could explain the lack of expression detected from ESTs, as lingual cDNA libraries are rarely included in large-scale sequencing projects. Finally, as with our method, RefSeq and ENSEMBL have also classified half of these regions as functional genes (see the list and the overlap at http://www.bork.embl.de/Docu/human_mouse_duplicates).

## Discussion

The identification and annotation of gene duplicates are of considerable relevance for several reasons: to uncover closely related genes with similar functions, to investigate the underlying mechanisms that modulate gene and protein families, and through comparative approaches, to identify changes in gene families associated with lineage specific traits. Although a large fraction of duplicated genes are included in a number of gene annotations, up to now there have been no global efforts directed to specifically resolve complex genomic regions with multiple gene copies and to distinguish genes and unprocessed pseudogenes. Therefore, we have performed the first comprehensive search in the human and mouse genomes of all duplicates of protein coding genes that are detectable through similarity to known proteins. Although not considering existing gene annotation, we recovered the vast majority of manually annotated genes (obtained from RefSeq), but also identified novel genes (222 in human and 198 in mouse), which constitute around 1% of the entire repertoire of protein coding genes in these organisms. We also obtained a comprehensive collection of unprocessed pseudogenes in human and mouse. The number of human and mouse unprocessed pseudogenes identified in this study are probably an accurate representation of the total number of these regions in this genomes, but we cannot discard the possibility that a fraction of them remained unidentified due to the conservative nature of our approach (e.g., a small fraction of the single-exon ones might have been labeled as processed).

Because we collected all (i.e., functional and dispensable) gene duplicates independently of their role in the organisms, we could uncover some mechanistic and evolutionary aspects of gene duplication that cannot be properly addressed by only considering the fraction of copies that were retained in those genomes either by genetic drift or by providing a selective advantage, i.e., functional genes: (1) We have observed that the vast majority (75%) of segmental gene duplications occur in tandem indicated by the location of recent duplicates (>95% sequence identity). With time, these copies tend to move to other locations, mostly to other chromosomes. From our analysis, it is not clear though whether this flow of gene copies is random or subject to some kind of pressure that favors some genes to remain together or apart, perhaps because they need to share or avoid common regulatory elements. A detailed analysis of the data provided here could reveal possible functional constraints behind these gene flows and could contribute to the understanding of the general rules that define the distribution of the genes in mammalian genomes. (2) We also found that the majority of genes are duplicated completely or nearly completely, probably as part of large duplicated segments [32]. The assignment of ESTs to a large fraction of gene duplicates suggests that promoter sequences are also often duplicated along with coding sequence. The fact that a large number of silenced coding regions have evidence of transcription demonstrates that these duplicated promoter sequences can remain operative despite the functional relevance of the transcribed region, contributing to the overall bulk of neutral transcription. A subset of human expressed pseudogenes has been also recently reported [33]. Even though we cannot discount the possibility that expressed coding regions that appear truncated could eventually act in the regulation of paralogs (possibly as antisense as previously observed [34]), these results question the relationship between transcription and functionality that is often assumed during gene annotation. (3) Our results also illustrate the plasticity of genes and proteins, as we uncovered the first examples of gene duplicates that have remained conserved in different mammalian lineages and therefore are likely to be functional although they appear to be considerably shorter than their close paralogs. Further investigation should reveal whether these copies are the result of partial gene duplication or are due to subsequent deletion events as recently suggested for the primate-specific RGP gene family [9].

The development of a protocol for the automatic annotation of genes and unprocessed pseudogenes in complex regions with many duplicated and often fragmented (pseudo)genes should also facilitate the annotation of other metazoan genomes. Here we show that the resolution of such regions deserves particular attention and cannot be solved using traditional approaches employed during primary genome analyses. Furthermore, we also provide preliminary novel biological insight into the mechanism of gene duplication and into the evolution and fate of gene duplicates, as well as the necessary data that will allow further and more detailed investigations in these directions.

## Materials and Methods

### Comparison with other gene and pseudogene sets.

We used the following reference datasets of genes and pseudogenes in order to assess our methology: RefSeq (http://www.ncbi.nlm.nih.gov/RefSeq), ENSEMBL (v36: http://www.ensembl.org), VEGA (v15: http://vega.sanger.ac.uk), and ENCODE (Dec 2005: http://www.genome.gov/10005107). For each set, we made clusters of transcripts and, in the case of splice variants, we selected the longest transcript per gene. The number of unique transcripts in these sets are 17,533 (RefSeq protein coding genes), 6,542 (RefSeq reviewed genes), 21,579 (ENSEMBL protein coding genes), 10,587 (VEGA protein coding genes), 434 (ENCODE protein coding genes), 704 (VEGA unprocessed pseudogenes), 71 (ENCODE unprocessed pseudogenes), 125 (ENCODE processed pseudogenes), 3,168 (VEGA processed pseudogenes), and 6,543 (Yale processed pseudogenes). For each of these regions, the coordinates on the genomic sequence are compared with those of the regions predicted in this study. We consider that there is overlap at genomic level between two regions of two sets if at least one nucleotide of the coding sequence (CDS) overlaps and both predictions are in the same strand.

### Identification and evaluation of gene duplicates, and calculation of *d*_S_.

In the context of identification of pseudogenes, we have previously already screened the human (build34) and the mouse (mm5, May 2004) genomes for all regions that showed significant sequence similarity to known proteins (EMBL CDS translations + SwissProt + RefSeq) as described elsewhere [10,14]. In brief, we have compared with BLAST all available known proteins against the human and mouse genomes and predicted the coding sequences (using GENEWISE) in all the regions that presented significant protein matches. Here we have used an updated version of the BLAST2GENE program [35], which efficiently identifies independent gene duplicates within complex multicopy regions. We finally accepted predictions only when we could unambiguously map them with BLAT [36] to the same chromosome on the finished human genome (build35) with more than 98% identity.

The evaluation of our procedure that identifies and reconstructs gene and derived coding regions was carried out using a jackknife test. For this we predicted the sequence of known RefSeq (reviewed) genes without using their own cDNA translation, but instead their closest sequence in the database (less than 98% identity). The predictions that we obtained for each of these genes were then compared with their real products at different levels in order to assess the coverage and the quality of our sequences. Note that the results of this evaluation tend to lower the real accuracy of our procedure as for many of the detectable regions in the genome, the final predictions are expected to be of higher quality because the real peptide product was used.

To make human and mouse orthologous predictions and thus to be able to discard retrotransposed regions, we have applied a two-step approach, based on sequence comparison and evaluation of genomic position. First, we have compared with BLASTP the translations of all human and mouse predicted regions (stop codons and frameshifts were circumvented for this comparison) and extracted up to 23,000 best reciprocal mouse–human hits that we took as candidate orthologous pairs. Of these, we only accepted as reliable ortholog pairs 17,164 that had other neighboring best reciprocal hits in both organisms. With this filter, we excluded those orthologous-looking pairs detected in which recently formed processed pseudogenes (almost identical to their parental gene) were likely to be involved (the majority of these pairs were formed by a single exon in one organism and an intron containing prediction in the other). We fixed the retained pairs and further compared with BLASTP each unassigned prediction found in-between with all predictions in the other species that were located in the corresponding orthologous region expanded 2 Megabases in both directions, and accepted as orthologs those sequences that showed a significant (Evalue < 0.001) match.

We estimated the rate of synonymous substitutions (*d*_S_) for each of the matching pairs of the set defined as segmental (i.e., non-retrotransposed) gene duplicates. For each of these pairs, we constructed a protein-based (CLUSTALW; [37]) DNA alignment. *d*_S_ values were calculated using *codeml* included in the *PAML* package [38]. We grouped all matching pairs through single linkage clustering, and sorted them chronologically by the UPGMA algorithm [39] using *d*_S_ values as distances in order to infer the order of each of the duplication events.

### Evaluation of the coverage of coding regions and estimation of P_f_.

To evaluate the coverage for a particular gene duplication, i.e., the relative length of the duplicated coding regions, we compared the length of each of the identified copies with each of their paralogs. If a group of genes underwent several rounds of duplications, we calculated the coverage of the older duplication events by comparing the lengths of the longest products at each of the resulting branches. Following the example shown in Figure 2A, we inferred the relative coverage of duplication “1” in human by comparing each of the resulting genes (“a” with “c” and “d,” and “b” with “c” and “d”) and selected the largest of all ratios obtained.

We applied the Bayes theorem to calculate the probability of being potentially functional for a given coverage, *P*(*f* | *c*). For this, we adopted two assumptions: (1) All human duplications that are orthologous to mouse are functional, and (2) all the duplications that formed complete coding regions (i.e., with coverage (*c*) = 1.0) are potentially functional. From the Bayesian law, the probability is calculated as:

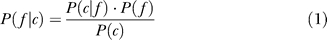
where *P*(*c*| *f*) is the fraction of orthologous duplications with a given coverage, and *P*(*c*) is the frequency of that coverage within all the duplications, which corresponds to the fraction of duplications with a certain coverage divided by the total number of duplications.

From the second assumption, Equation 1 can be re-written as:

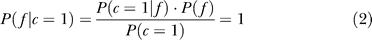
therefore,

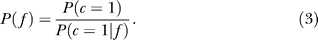
From Equations (1) and (3), we obtain:

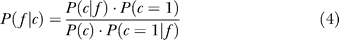
Hence, *P*(*f* |*c*) is calculated from *P*(*c*|*f*) and *P*(*c*), which are represented as,

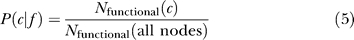
and

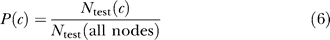
respectively, where *N*_functional (or test)_(*c*) corresponds to the number of orthologous duplications (or duplications under test) with a certain coverage *c*. We next calculated these functions by fitting an exponential curve to the observed data points (see Figure 2B) and obtained

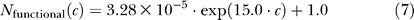
and

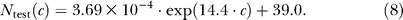



See Figure 2B for a graphical representation of the fitting exponential curve (Equation 7) corresponding to the functional cases.

From Equations 4, 7, and 8, we are finally able to calculate the probability of being potentially functional for any given duplicate according to its coverage using the final equation (Equation 9):


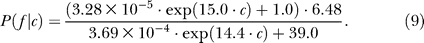


### Mapping of human ESTs.

All ESTs from dbEST (http://www.ncbi.nlm.nih.gov/dbEST; as of February 2004) were aligned against the human genome using stand-alone BLAT [36], and we accepted only those matches with a percentage identity greater than 96% and with an alignment length greater than 100 bases. If the difference in score between the best hit and second-best hit was less than ten in a BLAT-like scoring scheme, we considered such an EST alignment as ambiguous and excluded it from our study.

(Supplementary data can be found at http://www.bork.embl.de/Docu/human_mouse_duplicates.)

## Supporting Information

Figure S1 Distribution of Human Gene Duplications According to the Degree of Protein Identity among Their Gene Products, and Their Present Location in the Genome (in the Same or Different Chromosomes) (494 KB PDF)

Figure S2 Example of Functional Partial Tandem Duplicate (437 KB PDF)

### Accession Numbers

The GenBank (http://www.ncbi.nlm.nih.gov/Genbank) accession numbers for the genes discussed in this paper are human ETS-2 repressor factor gene *(ERF)* (50403684), human Sp5 transcription factor gene (51460866), and mouse Sp5 transcription factor gene (9454416).

The RefSeq (http://www.ncbi.nlm.nih.gov/RefSeq) accession number for the copy of the gene encoding the ETS-2 repressor factor *(ERF)* is NP_006485.1.

## Abbreviations

EST: expressed sequence tag
