# Supplementary material for: Identification and Analysis of Genes and Pseudogenes within Duplicated Regions in the Human and Mouse Genomes
Source: PLoS Comput Biol. 2006 Jun 30;2(6):e76. doi: 10.1371/journal.pcbi.0020076 (PMC1484586; doi:10.1371/journal.pcbi.0020076)
Supplement: Figure S1 — (494 KB PDF) [file pcbi.0020076.sg001.pdf]

Figure S1

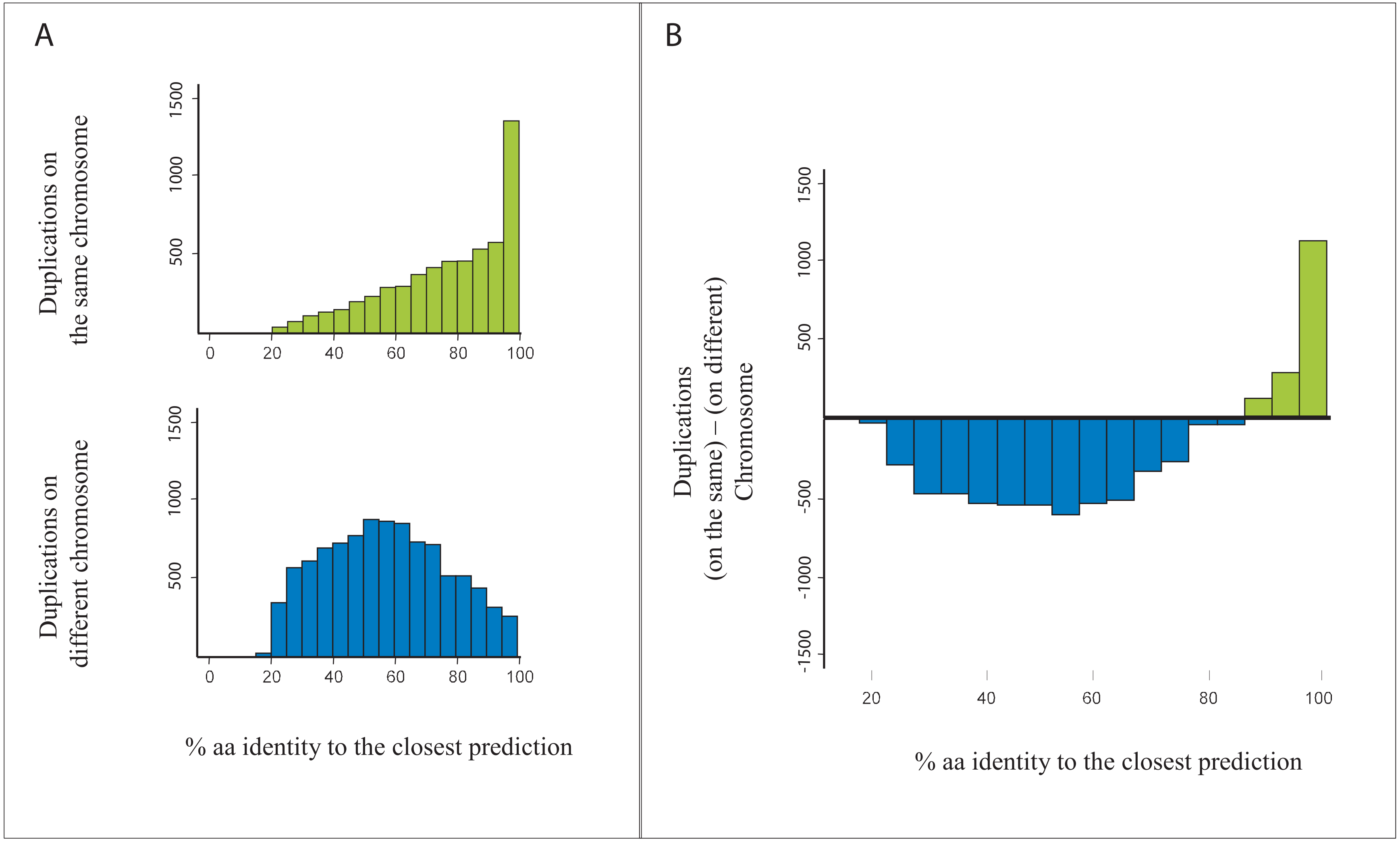

Figure 1. Supp. **Distribution of human gene duplications according to the degree of protein identity among their gene products, and their present location in the genome (in the same or different chromosome).** Please note that the protein identity for older duplications, whose products might have undergone successive duplication, corresponds to the averaged identity of all products and derived duplicates.
