# Supplementary material for: Identification and Analysis of Genes and Pseudogenes within Duplicated Regions in the Human and Mouse Genomes
Source: PLoS Comput Biol. 2006 Jun 30;2(6):e76. doi: 10.1371/journal.pcbi.0020076 (PMC1484586; doi:10.1371/journal.pcbi.0020076)
Supplement: Figure S2 — (437 KB PDF) [file pcbi.0020076.sg002.pdf]

Figure2 (supp)

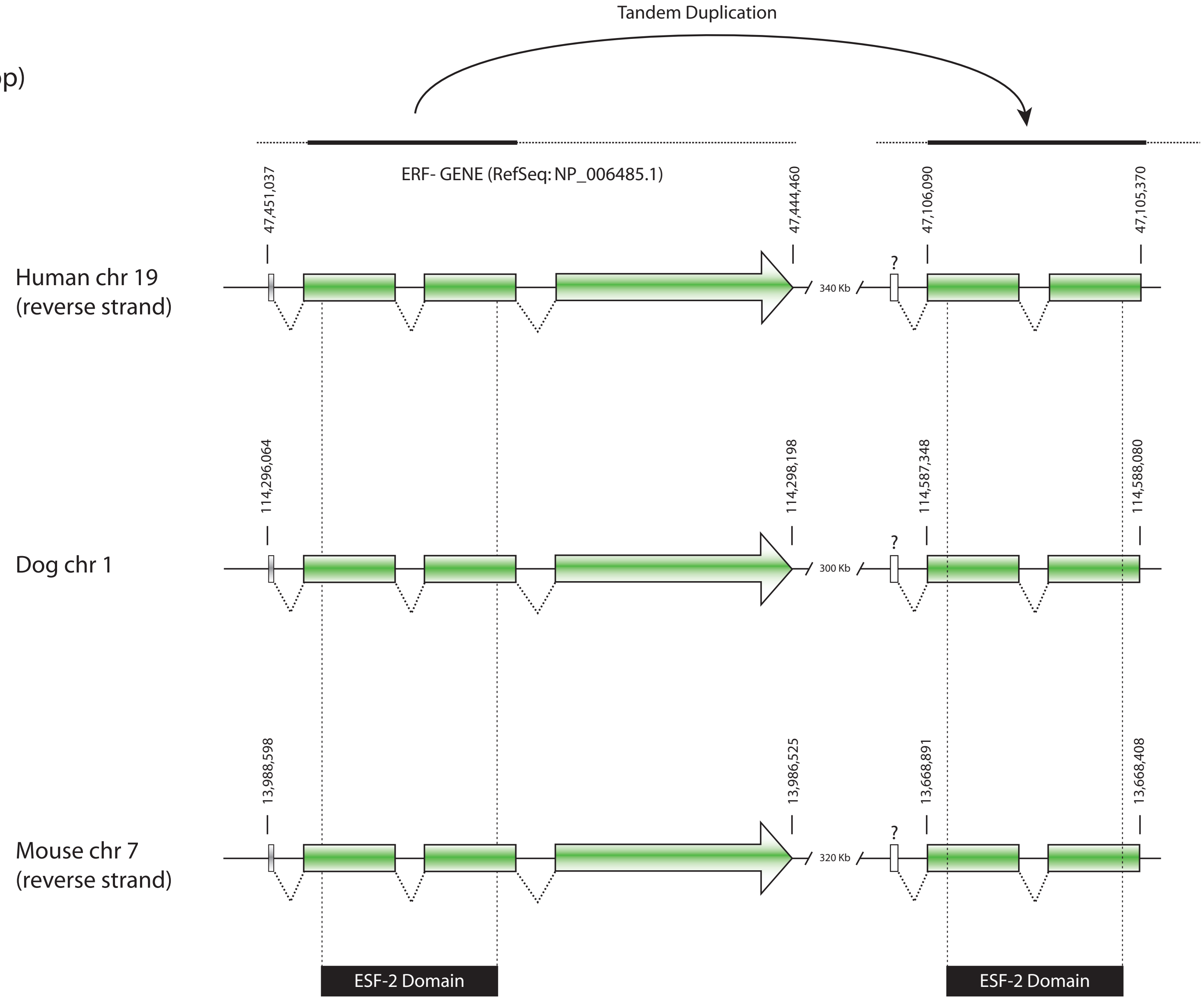

Figure S2. **Example of functional partial tandem duplicate.** The orientation of the gene is displayed on the reverse strand. Exon and intron sizes are not in scale. See text for details.
